# Next-Generation Drug Delivery for Neurotherapeutics: The Promise of Stimuli-Triggered Nanocarriers

**DOI:** 10.3390/biomedicines13061464

**Published:** 2025-06-13

**Authors:** Radka Boyuklieva, Nikolay Zahariev, Plamen Simeonov, Dimitar Penkov, Plamen Katsarov

**Affiliations:** 1 Department of Pharmaceutical Technology and Biopharmacy, Faculty of Pharmacy, Medical University of Plovdiv, 4002 Plovdiv, Bulgaria; radka.boyuklieva@mu-plovdiv.bg (R.B.); nikolay.zahariev@mu-plovdiv.bg (N.Z.); plamen.simeonov@mu-plovdiv.bg (P.S.); dimitar.penkov@mu-plovdiv.bg (D.P.); 2 Research Institute at Medical University of Plovdiv (RIMU), 4002 Plovdiv, Bulgaria

**Keywords:** stimuli-responsive nanocarriers, neurodegenerative disorders, targeted delivery

## Abstract

Nanotherapeutics have emerged as novel unparalleled drug delivery systems (DDSs) for the treatment of neurodegenerative disorders. By applying different technological approaches, nanoparticles can be engineered to possess different functionalities. In recent years, the developed, stimuli-responsive nanocarriers stand out as novel complex DDSs ensuring selective and specific drug delivery in response to different endogenous and exogenous stimuli. Due to the multifaceted pathophysiology of the nervous system, a major challenge in modern neuropharmacology is the development of effective therapies ensuring high efficacy and low toxicity. Functionalization of the nanocarriers to react to specific microenvironmental changes in the nervous system tissues or external stimulations significantly enhances the efficacy of drug delivery. This review discusses the microenvironmental characteristics of some common neurological diseases in-depth and provides a comprehensive overview on the progress of the development of exogenous and endogenous stimuli-sensitive nanocarriers for the treatment of Alzheimer’s and Parkinson’s disease.

## 1. Introduction

**Figure 1:**
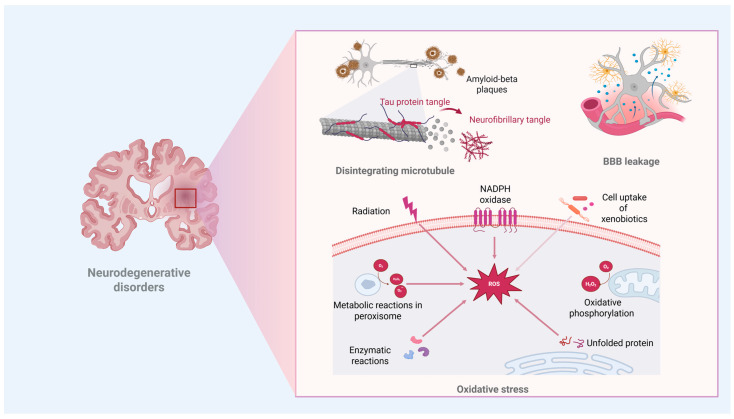
Some pathological mechanisms related to neurodegenerative disorders (Created in BioRender. https://BioRender.com/wnz652y). Note: The arrows indicate different mechanisms for ROS generation.

According to the World Health Organization, neurological disorders may become the second leading cause of mortality within the next two decades [1]. The increasing number of cases of neurodegenerative diseases (NDs) in the aging population drives biomedical researchers to broaden their investigation on conditions such as Alzheimer’s disease (AD), Parkinson’s disease (PD), dementia, amyotrophic lateral sclerosis, multiple sclerosis, and others [2]. NDs lead to the gradual decline of brain neurons. Each condition has unique characteristics. However, they show some common expressions, such as protein misfolding, which results in toxic aggregates (Figure 1). For example, AD is characterized by amyloid-beta plaques and tau tangles [3,4,5], PD involves alpha-synuclein aggregates [6,7], and prion diseases like Creutzfeldt–Jakob disease are associated with prion protein aggregates [8,9].

Neurons are particularly vulnerable to oxidative stress due to their high energy demands and low levels of antioxidant enzymes. This vulnerability can damage lipids, proteins, and DNA, eventually leading to cell death [10,11,12]. Mitochondria play a crucial role in generating energy for neurons; when they malfunction, it results in decreased ATP levels, increased levels of reactive oxygen species (ROS), and the activation of apoptotic processes [13,14]. Additionally, microglia-induced chronic inflammation can contribute to neurodegeneration by releasing harmful pro-inflammatory cytokines [15,16]. Furthermore, excessive activation of glutamate receptors may lead to excitotoxicity, causing calcium influx and a cascade of damaging events, including mitochondrial dysfunction and apoptosis [17]. Injuries can exacerbate ongoing neurodegenerative processes and elevate systemic neuroinflammatory responses by affecting the central nervous system (CNS) [18,19].

A significant challenge facing modern therapy for neurodegenerative diseases is ensuring the effective passage of the therapeutic agent through the blood–brain barrier (BBB) [20].

Many medicinal substances have a low solubility and inadequate transport across the BBB, which can affect their therapeutic effectiveness. Although various surgical techniques and invasive methods have shown some success, their clinical acceptance and application are limited due to the risk of potential long-term side effects and possible damage to the BBB. Currently, most macromolecular drugs and some small drug molecules are unable to cross the BBB successfully, resulting in poor biopharmaceutical and pharmacokinetic characteristics [21]. In order to overcome these limitations, modern pharmaceutical therapy is focused on the development of new technological approaches, ensuring optimal drug delivery through the BBB and thus achieving effective therapy. One promising strategy is the use of nanotherapeutics which are capable of easily crossing the BBB [22].

Nanoscale drug delivery systems are multiparticulate systems composed of particles ranging from 10 to 1000 nm in size. Drug substances are dissolved, dispersed, or adsorbed onto the surface of a suitable carrier [23]. Unlike conventional dosage forms, nanosized systems demonstrate significantly enhanced pharmacokinetic and pharmacodynamic properties of the encapsulated drug. Many drug substances cannot easily cross the biological membranes and exhibit low bioavailability in the body, attributed to their poor solubility and limited stability in physiological environments [24]. Due to their small size, most nanoparticles (NPs) can pass through the biological membranes via endocytosis, while some may also utilize paracellular transport [25,26]. Engineering the NPs to have the appropriate shape and size (<200 nm) can allow their effective navigation across the tight junctions between endothelial cells of the BBB. Generally, spherical and elongated particles exhibit improved penetration capability [27]. Coating NPs with biocompatible materials like polyethylene glycol (PEG) can help them evade the immune response and reduce clearance by macrophages (a technique known as stealth technology) [28,29]. For instance, in a mouse model study, lactoferrin-conjugated PEG-PLA NPs improved the transport of the conjugated particles to the brain [30]. Moreover, modifying the surface of NPs with ligands that bind to specific receptors on the BBB can enhance receptor-mediated endocytosis [31,32].

In recent years, there has been significant progress in the development of stimuli-responsive nanocarriers aimed at delivering therapeutic agents and diagnostic tools. Responding to specific stimuli, such systems can facilitate achieving more effective therapeutic effects.

**Figure 2:**
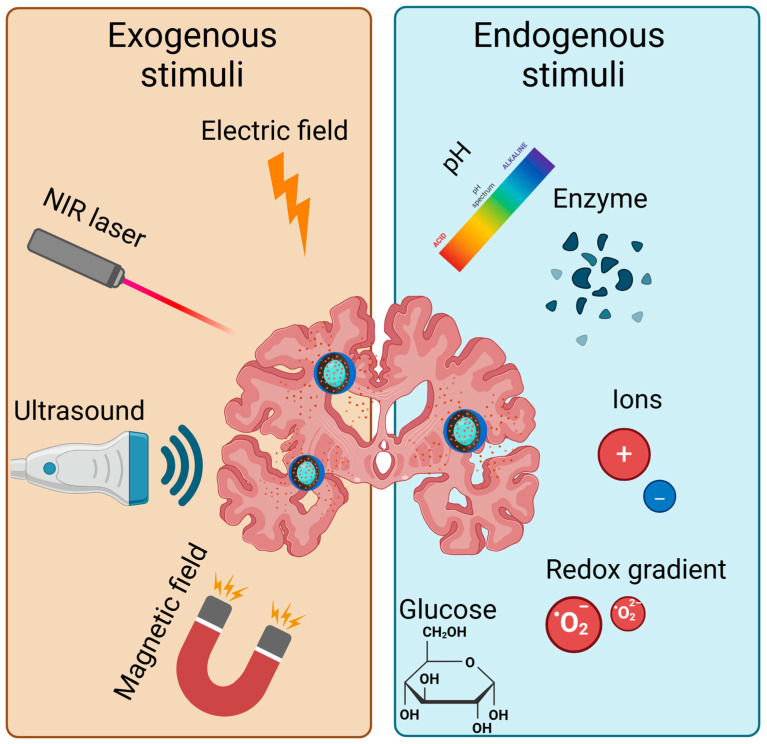
Exogenous and endogenous stimuli which can affect drug delivery (Created in BioRender. https://BioRender.com/ohosvgj).

Stimuli-responsive drug delivery primarily falls into two categories: endogenous (including changes in pH, enzymatic activity, redox gradients, ions, and glucose) and exogenous (including light, ultrasound, electric fields, and magnetic fields) (Figure 2). These smart nanocarriers can adjust their behavior in response to the environment, allowing enhanced control over drug release and improved therapeutic outcomes [33].

This review article examines the recent developments of stimuli-responsive nanosized drug delivery systems providing enhanced treatment of neurodegenerative disorders. It is focused mainly on Alzheimer’s and Parkinson’s disease, as two of the most prevalent neurodegenerative disorders worldwide, being of high social and medical significance, particularly in aging populations, and having a profound impact both on patients and the healthcare system. Although these neurodegenerative diseases have been extensively studied, their treatment is still a challenge, highlighting the urgent need for innovative therapeutic strategies. In this context, novel drug delivery systems are continually being developed, offering promising new approaches for more effective treatment.

Moreover, both Alzheimer’s and Parkinson’s disease have been the focus of intensive research efforts aimed at developing stimuli-responsive nanotherapeutics. Although such approaches are still at the preclinical stage, these diseases present well-characterized pathological environments that could potentially be exploited by smart drug delivery systems, making them relevant models for reviewing the current progress and challenges in this emerging field. The literature analyzed in this review includes research articles from recent years, highlighting the current advancements in the therapy of AD and PD using novel stimuli-triggered nanotherapeutics.

## 2. Concepts Behind Stimuli-Responsive Drug Delivery in Neurodegenerative Diseases

### 2.1. Drug Delivery Systems, Triggered by Endogenous Stimuli

#### 2.1.1. pH-Responsive Approaches

The pH level is commonly used as a drug delivery trigger for specific organs, such as the gastrointestinal tract [34], or organelles like lysosomes, endosomes, and the Golgi apparatus [35]. It can also trigger the release of active molecules under modified pathological conditions, such as cancer, inflammation, or ischemia, characterized by significant pH variations. In healthy brain tissue, the intracellular pH typically falls in the range 7.1–7.3, while the extracellular pH is slightly acidic, usually ranging from 6.8 to 7.2 [36]. Persistent inflammation, often associated with neurodegenerative diseases, can increase lactic acid production due to heightened glycolysis [37,38]. This process can lower pH levels, resulting in a more acidic environment [39,40]. Although specific pH levels may vary based on the type and stage of the NDs, it is commonly established that affected brain regions become more acidic.

**Figure 3:**
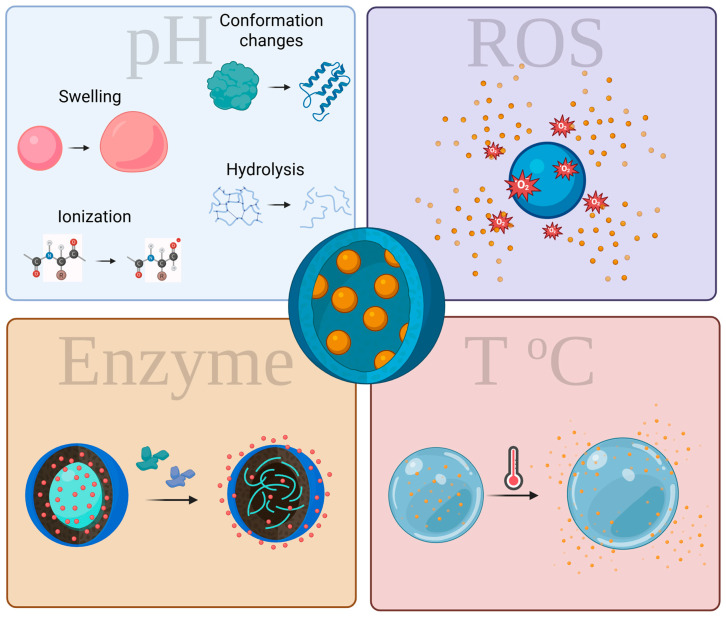
Endogenous stimuli-sensitive mechanisms for drug delivery (Created in BioRender. https://biorender.com/0svue7j).

Therapeutic systems designed to respond to stimuli have been developed to maintain their stability in healthy tissues while releasing their cargo in diseased areas that exhibit structural changes due to pH fluctuations [41]. pH-sensitive NPs react to changes in pH through four mechanisms: (i) the protonation or ionization of functional groups, (ii) the hydrolysis of acid-labile bonds, (iii) conformation changes, and (iv) the swelling of NPs (Figure 3) [42]. A crucial mechanism is the protonation reaction, where variations in hydrogen ion (H^+^) concentration lead to changes in the charge of NPs. For example, polymers containing carboxylic acid (-COOH) or amine (-NH_2_) groups can modify their charge based on the pH levels of the surrounding medium. In acidic conditions, carboxylic groups become protonated and lose their negative charge, while in alkaline conditions, they deprotonate and regain a negative charge. These reactions can cause changes in the structure, size, and properties of the NPs [43].

Hydrolysis is another process which can lead to NP disintegration or alteration due to their interaction with water, which is influenced by pH levels. This may result in modifications to the structure and characteristics of NPs. For instance, certain esters, amides, imines, and ketals can undergo hydrolysis at specific pH levels, releasing active compounds or altering the shape of the NPs [44,45,46]. pH-sensitive NPs include polymers or other molecules that can change their structure based on pH levels [47]. For instance, polymers with pH-responsive side chains can perform a transition from a coiled to an extended structure when the pH changes, resulting in variations in the size and characteristics of the NPs.

Drug release can be controlled through swelling mechanisms using pH-sensitive nanoparticle micelles, which are constructed with hydrophobic segments linked by acid-sensitive bonds that enable self-assembly in water at a physiological pH level. In acidic environments, such as inflamed tissues, H^+^ ions penetrate the micelles, causing the bonds there to degrade and the hydrophobic segments to break down. This leads to an increase in solubility and the expansion of micelles, enhancing drug release [48]. A typical approach involves attaching 2,4,6-trimethoxybenzaldehyde to the hydrophobic segments via acetal bonds; in acidic conditions, the hydrolysis of the acetal results in significant micelle swelling due to a decrease in hydrophobicity [49,50].

#### 2.1.2. Oxidation-Responsive Approaches

Several neuropathological conditions are characterized by an excessive production of ROS due to inflammatory responses [51]. Over the years, strategies to address neurological disorders have often focused on antioxidants and pharmaceuticals that inhibit specific cellular receptors and signaling pathways. However, many therapies do not effectively target neuroinflammation, causing these medications to circulate throughout the body. This can result in the need for higher drug concentrations, leading to adverse side effects such as overdoses, oxidative stress in localized areas, and unintended reactions in other parts of the body.

To address this issue, drug carriers can be engineered as nano-sized particles that respond to elevated levels of ROS. These particles can release their contents rapidly when exposed to high ROS concentrations [52,53]. The materials incorporated within these particles can range from small compounds to larger biomacromolecules. These agents can target pathways associated with neuropathology or help reduce inflammation by suppressing inflammatory pathways or neutralizing ROS. Such molecules include antioxidants, anti-inflammatory medications like dexamethasone [54] and epigallocatechin gallate, and drugs designed explicitly for neurological diseases, such as resveratrol [55,56].

Researchers nowadays can offer approaches for particle design, which allow the attachment of chemicals that interact with ROS. These interactions can modify properties such as charge and hydrophilicity, cause bond cleavage, or trigger reactions that result in particle expansion, disassembly, or enhanced drug diffusion. Depending on the intended outcome, specific mechanisms can be selected. For instance, nanoparticles that swell and gradually release drugs in response to ROS are ideal for managing inflammation. In contrast, others may generate oxygen gas from hydrogen peroxide (H_2_O_2_) to facilitate rapid drug release. Careful selection of chemical components is crucial to achieving the desired effects in drug delivery [57,58].

#### 2.1.3. Enzyme-Responsive Approaches

A key area of research in stimuli-responsive “smart” nanomaterials focuses on developing materials that react to enzyme activity [59]. Enzymes play a crucial role in biological functions, and their dysfunction is linked to various diseases [60,61]. Utilizing enzymes as biological triggers offers several advantages: they catalyze reactions under mild conditions (such as neutral pHs or high temperatures) and provide high selectivity for specific substrates, allowing for precise chemical transformations [62].

Nanomaterials designed to be sensitive to enzyme activity include components that enzymes can break down [63]. Various nanoscale materials—such as polymers, phospholipids, and inorganic compounds—have recently been employed in drug delivery systems that respond to enzymes. These nanomaterials provide bio-specificity and selectivity, enabling innovative applications like nanoparticles that actively target organs using enzyme-activated components. This strategy enhances drug concentration at specific sites, minimizes uptake by non-target tissues, and allows for controlled drug release while maintaining targeting efficacy.

Enzyme responsiveness is achieved through moieties in the main chain or side groups that can be cleaved by specific enzymes. For example, self-assembled nanoparticles often incorporate enzyme-responsive linkers to regulate cargo release. Additionally, inorganic nanosystems can be enhanced by adding active-targeting ligands that react to enzymes, broadening their design possibilities and potential applications [62,63].

#### 2.1.4. Thermo-Responsive Approaches

Living organisms have cells that can withstand temperature fluctuations from freezing to about 42 °C without sustaining damage. However, pH levels and ionic strength must remain within specific limits to maintain cellular viability [64]. Various thermo-responsive polymer nanocarriers, including hydrogels and nanoparticles, have been developed. The widely used thermo-responsive polymers have phase transition temperatures that fall within the range that physiological conditions can tolerate.

Polymers featuring a lower critical solution temperature (LCST), such as poly(N-isopropylacrylamide) (PNIPAAm), are commonly employed due to their ability to switch from a hydrophilic to a hydrophobic state when temperatures exceed their LCST of 32 °C [65,66,67,68]. This phase transition results in the breakdown of the nanocarriers, promoting faster drug release. However, there are concerns regarding the biocompatibility of PNIPAAm, as it is less desirable because of the neurotoxic effects associated with acrylamide monomers and the potential for toxic amine compounds, formed when PNIPAAm degrades in acidic environments [69].

Other polymeric materials, like poly(γ-2-(2-(2-methoxyethoxy)-ethoxy)ethoxy-ε-caprolactone)-b-poly(γ-octyloxy-ε-caprolactone), also demonstrate LCST characteristics, enabling rapid drug release under hyperthermic conditions [70]. The transition temperatures of these polymers can be modified by adjusting factors such as the polymer chain length, branching, and the ratio of hydrophilic to hydrophobic components. By carefully tuning these properties, thermo-responsive polymers can be engineered as nanocarriers that facilitate drug-controlled release in response to temperature changes [71].

### 2.2. Drug Delivery Systems Triggered by Exogenous Stimuli

**Figure 4:**
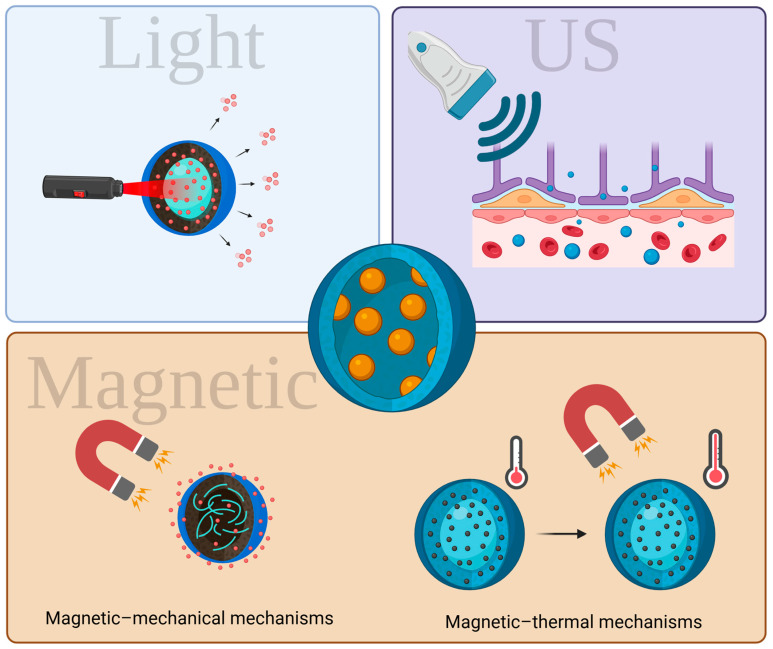
Exogenous stimuli-sensitive mechanisms for drug delivery (Created in BioRender. https://BioRender.com/otmw5mt).

Alternative methods for enhanced drug delivery involve systems which can respond to external stimuli (Figure 4). Optical stimulation offers high spatial and temporal resolution but has limited penetration depth (approximately 1.5 mm) [72]. Acoustic stimulation, primarily through focused ultrasound (FUS), can reach deeper tissues (up to 450 mm). However, it has lower spatial resolution (about 41 mm^3^) and temporal accuracy (410 ms) [73]. Non-invasive magnetic fields can effectively target deep brain areas, making them a promising option for treating neurological conditions [74,75]. For example, o-nitrobenzyl compounds are used for light-activated release [76], while gold nanoparticles facilitate photothermal release [77]. Micro- and nanobubbles are commonly employed for ultrasound delivery, and iron oxide NPs are utilized for magnetic–thermal release. Despite these advancements, delivering neurochemicals to the brain remains challenging due to the complex microenvironment and low active concentrations. Significant progress has been made in developing various drug delivery systems that respond to external neuromodulation stimuli, outlined below.

#### 2.2.1. Light-Responsive Approaches

The expansion of spectral absorption into the near-infrared (NIR) region, particularly NIR-II (1000–1700 nm), has significantly enhanced the stimulus-responsive materials. This advancement allows deeper tissue penetration and minimizes phototoxic effects during neuroimaging and neuromodulation [78]. Light-driven neuromodulation enables the release of neuroactive substances primarily through photolysis, using caged compounds that protect bioactive molecules until they are activated by light. For example, Banghart et al. engineered light-sensitive variants of the mu-opioid receptor agonist oxymorphone and the antagonist naloxone, enabling precise modulation of neural circuits with fewer adverse effects [79,80]. They also introduced a biomimetic caging technique to improve the effectiveness of neuropeptides [81].

Advancements in DNA technology have led to the development of DNA-based materials that can self-organize into structures through Watson–Crick base pairing [82]. These structures can release drugs in response to various stimuli by employing molecular switches. Kohman et al. demonstrated DNA-based structures for light-triggered release, resulting in Ca^2+^ signaling in hippocampal neurons [83]. Photoisomerization allows specific molecules, such as azobenzene, to change their shape in response to light. Azobenzene exists in a stable *trans* form and a metastable *cis* form; exposure to UV light converts it to the *cis* form, which can revert to the *trans* form under visible light. This property benefits various molecular devices and has enabled precise modulation of glutamate receptors in neurons via two-photon excitation [84]. “Photolipids,” including azobenzene, can alter the properties of lipid bilayers when exposed to light. A notable derivative, azo-PC, changes its structure in response to light, affecting membrane permeability and facilitating controlled drug release [85]. Recent research on “azosome” nanovesicles containing azo-PC suggests that they allow for accurate control of neuronal activity and rapid material release at specific light wavelengths, all while minimizing heat and ROS, thus enhancing safety in neuromodulation [86]. Photothermal triggered release utilizes materials that absorb light and convert it into heat, increasing temperature, which assists in releasing encapsulated neuroactive agents. For instance, Li et al. developed a drug delivery system using conjugated polymer NPs that release fasudil to modulate ion channels associated with depression when exposed to NIR light. This system achieves a photothermal conversion efficiency of 57.48% when irradiated with a laser at 808 nm, allowing the NPs to reach temperatures between 40–42 °C within 50 s. These NPs also effectively cross the BBB and decrease the firing of dopamine neurons linked to depression [87]. Furthermore, Kohane et al. employed gold nanorods linked to thermosensitive liposomes for the controlled release of tetrodotoxin under NIR light. This method offers prolonged local anesthesia while improving safety. Gold nanorods in low-temperature-sensitive liposomes enhance light absorption, reduce thermal toxicity, and accelerate drug release [88].

#### 2.2.2. Ultrasound-Responsive Approaches

Ultrasound stimulation provides a non-invasive alternative to surgical methods such as deep brain and spinal cord stimulation, which carry surgical risks. FUS stimulation can target specific areas within the brain with high spatial accuracy (1–2 mm^3^) and reach deeper structures than transcranial electrostimulation or transcranial magnetic stimulation [73,89]. This capability is valuable for exploring neural circuits and treating neurological conditions. Additionally, sonogenetics merges sound stimulation with genetically modified ion channels to influence specific neurons [90]. Recent advances in nanoparticle technology and FUS modulation have increased interest in sonomechanically mediated neuromodulation, particularly for targeted drug delivery across the BBB. Airan et al. discovered that transcranial FUS stimulation can induce a phase transition in perfluorocarbon-based nanoemulsions. This transition enables propofol, a fat-soluble drug, to effectively cross the BBB when sonicated at 1 MHz. This method allows phase-change NPS to encapsulate hydrophobic small molecules [91]. Lea-Banks et al. utilized ultrasound-responsive nanodroplets to release pentobarbital for local anesthesia in rats while maintaining BBB integrity. These nanodroplets have advantages as drug carriers, including limited systemic distribution and controlled release [92]. Ozdas et al. introduced an innovative ultrasound-controllable drug carrier that combines aggregation and caging with an FUS sequence. This approach allows for precise drug delivery and modulation of sensory signaling with minimal side effects [93].

#### 2.2.3. Magnetic-Responsive Approaches

Magnetic fields are practical external triggers for targeted drug release without requiring direct interaction with patients. These techniques utilize magnetic NPs that convert magnetic fields into signals for biological receptors [94,95]. They can be categorized into two main types: magnetic–thermal, which generate heat, and magneto-mechanical mechanisms, which produce force (Figure 4). In the magnetic–thermal mechanism, alternating magnetic fields are mainly used alongside magnetic NPs to release neuromodulators. The heating efficiency is influenced by the characteristics of magnetic NPs, particularly their size and structure, with particles smaller than 50 nm being particularly effective. For instance, Guntnur et al. developed magnetic NPs coated with thermo-responsive poly(oligo(ethylene glycol) methyl ether methacrylate (POEGMA) brushes that release dopamine when heated by changing magnetic fields. This technique significantly enhanced the activity of the treated neurons, allowing for controlled and reversible drug release. It effectively regulates local heating, preventing the degradation of the drug and enabling control of ligand–receptor interactions [96].

Magnetic–mechanical NPs have the capability to manipulate mechanical forces at the cellular scale using low-frequency alternating magnetic fields while avoiding heat generation. The technique of magneto-mechanics shows potential for directing a range of molecules and structures, such as DNA, proteins, and lipid membranes. The effectiveness of this method relies on the morphology, size, and composition of the magnetic NPs, in addition to the amplitude, frequency, and arrangement of the magnetic field [97].

## 3. Stimuli-Responsive Nanotherapeutics for the Treatment of Neurodegenerative Disorders

### 3.1. Stimuli-Triggered Nanotherapeutics for Alzheimer’s Disease

Alzheimer’s disease (AD) is a complex neurodegenerative condition characterized by the presence of amyloid-beta plaques, tau tangles, oxidative stress, and ongoing neuroinflammation [98]. Due to the intricate nature of this disease and the challenge of crossing the BBB, there is an urgent need for the development of innovative smart drug delivery systems [99]. Stimuli-responsive nanocarriers, lipid- or polymer-based structures that react to specific stimuli, offer a promising solution, ensuring the effective crossing of biological barriers, thus providing specific and selective drug delivery to the targeted site [60,100,101,102]. The mechanisms underlying AD progression involve several biochemical changes that advanced drug delivery systems can exploit. These changes include an increase in endosomal pH and the pH of inflamed tissues, elevated levels of ROS near amyloid-beta plaques, and the overexpression of disease-related enzymes such as matrix metalloproteinases (MMP) and phospholipases [102]. Stimuli-responsive nanocarriers are designed to detect and respond to these microenvironmental changes, allowing localized drug release and improved therapeutic specificity (Table 1). Redox-responsive polymers that contain thioketal bonds, disulfide bridges, or phenylboronic esters can undergo bond cleavage or backbone fragmentation when exposed to ROS or glutathione (GSH), resulting in controlled drug release or nanoparticle disassembly [103].

Stimuli-responsive liposomes can deliver a wide range of therapeutic agents tailored to the multifaceted pathophysiology of AD. Anti-amyloid agents such as curcumin [104], clioquinol [105], and peptide inhibitors are delivered to disrupt amyloid-beta aggregation and promote plaque clearance [106]. Fernandes et al. developed a new class of liposomes—exosome-like liposomes (exo-liposomes)—that combine the natural bioactivity and brain-targeting capabilities of exosomes with the manufacturability and scalability of synthetic liposomes. This system was created to deliver curcumin, a hydrophobic compound known for its anti-amyloid, antioxidant, and anti-inflammatory properties. These particles demonstrate a size of less than 200 nm (ideal for BBB penetration), high encapsulation efficiency (up to 94%), and stability for 3–6 months when refrigerated [104]. Revdekar et al. synthesized pH-sensitive pullulan acetate to create silibinin-loaded nanoparticles for brain-specific delivery. Silibinin, a natural agent, inhibits amyloid-beta aggregation and reduces cognitive dysfunction in AD models. The optimized NPs had a particle size of 293.50 nm, a zeta potential of −5.82 mV, and an entrapment efficiency of 88.74%. The NPs exhibited a pH-sensitive release profile, with 34.61% release at pH 6.0 and 90.96% release at pH 7.2 over 24 h. Ex-vivo nasal mucosa studies demonstrated 85.84% drug permeation within 24 h, while brain uptake studies in rats indicated sustained release with significant concentrations over time [107]. In another study, Liu et al. described a dendrimer–peptide conjugate that responds to ROS for targeted delivery to the AD microenvironment, aiming to reduce early-stage inflammatory responses. This nanosystem was created using three components: a peptide, an ROS-responsive ethylene glycol-based boronic dendrimer with clearing properties, and a therapeutic peptide derived from nuclear factor (erythroid-derived 2)-like 2 (Nrf2). The developed nanosystem could cross the BBB and bind to the receptor for advanced glycation end products abundantly present in the AD microenvironment. It exhibited a combined effect that enhanced antioxidant efficiency and reduced glial cell reactivity by eliminating ROS and releasing Nrf2. Both in vitro and in vivo research demonstrated that suppressing inflammatory responses has neuroprotective benefits during the early phases of AD. Overall, this indicates that multi-target therapies could lead to better therapeutic outcomes in the initial stages of AD than single therapies, potentially offering greater prospects for clinical application [108].

In contrast, pH-sensitive nanocarriers utilize acid-labile bonds or fusogenic lipids to destabilize acidic lysosomes, enhancing endosomal escape and cytoplasmic drug delivery. These pH liposomes need to be precisely engineered at both the lipid and surface levels to enable targeted delivery and stimulated release. Key modifications may include using stimuli-sensitive lipids, PEGylation for prolonged circulation, and pH- or ROS-cleavable linkers to facilitate payload release [109]. For example, Gu et al. discovered that PEG-coupled liposomal NPs improve the water solubility and effectiveness of astaxanthin (ATX), a potent exogenous antioxidant, in alleviating cognitive impairments associated with AD. These nanoparticles, designed to deliver ATX to the brain, demonstrated reduced neurotoxicity by degrading formaldehyde and inhibiting amyloid-beta aggregation. In APPswe/PS1dE9 mice, administering PEG-ATX NPs intraperitoneally resulted in decreased levels of formaldehyde and malondialdehyde in the brain, lowered amyloid-beta oligomerization, and reduced the formation of senile plaques [110].

As mentioned above, surface engineering is crucial for crossing the BBB and enhancing cellular uptake. This often involves using ligands such as transferrin, lactoferrin, or glutathione to target specific receptors on brain endothelial or neuronal cells [111]. Transferrin-conjugated liposomes loaded with vitamin B12 effectively target the BBB and neuronal cells that have high transferrin receptor (TfR) expression. These liposomes, measuring under 200 nm and possessing neutral zeta potential, are well-suited for delivering substances to the brain. However, the hydrophilicity and high molecular weight of vitamin B12 have limited its anti-amyloidogenic effects in AD. Recent research suggests that Tf-modified liposomes can transport vitamin B12 to the brain and provide sustained release for up to 9 days, helping to delay the formation of amyloid-beta fibrils by preventing fibrillation and breaking apart existing fibrils [112]. Tf-modified liposomes can also incorporate various molecules that offer additional therapeutic and cytoprotective benefits, including anti-inflammatory and antioxidant effects [113,114,115]. Liposomes linked to apolipoprotein E (ApoE) and phosphatidic acid (PA) have shown potential in targeting amyloid-beta plaques in the brain and improving BBB penetration [116]. PA, which carries a negative charge, adheres to amyloid-beta plaques, while ApoE’s positive charge interacts with the negatively charged regions of beta-amyloid, promoting effective targeting [117]. Additionally, liposomes carrying ApoE2 plasmids have been developed with penetratin and mannose ligands for efficient transfection of brain cells, demonstrating therapeutic promise in restoring neuroprotective protein expression [118]. A cationic liposomal formulation of artesunate—a malaria medication with potential neurotherapeutic applications—was designed to cross the BBB and provide anti-inflammatory effects by targeting both canonical and non-canonical inflammasome pathways in a mouse model of sporadic AD. This formulation demonstrates pH-sensitive drug release, exemplifying stimuli-responsive liposomal nanocarriers in the context of AD [119]. Moreover, a biomimetic ApoE-reconstituted high-density lipoprotein nanocarrier has been developed to enhance BBB permeability and target amyloid-beta, allowing for the delivery of α-Mangostin to lower amyloid levels and improve cognitive function in animal models. This nanocarrier showed improved absorption in brain endothelial cells and higher delivery efficacy [120]. Furthermore, liposomes modified with an ApoE-derived peptide and PA increased calcium levels in human microvascular endothelial cells, providing protective effects against neuroinflammation induced by amyloid-beta [121]. Additional research has highlighted the advantages of PEGylation and ApoE3 coatings in liposome delivery, particularly for rivastigmine hydrogen tartrate. This formulation enhanced brain uptake and prolonged the circulation time of the drug [122]. Phosphatidylcholine (PC) liposomes fused with GSH and ApoE were studied for their ability to deliver various phytochemicals. This combination improved BBB permeability and drug release, suggesting its potential to enhance drug delivery to the brain [123].

Recent advancements in therapy are now focusing on dual stimuli-responsiveness, which allows more precise control over therapeutic actions [124]. For instance, Conti et al. functionalized liposomes with PA, modified ApoE, and a curcumin derivative. Their multifunctional system, comprising curcumin–lipid liposomes, demonstrates capabilities such as penetrating the BBB, binding to amyloid-beta, and facilitating controlled drug release [125].

In another study, Sokolik et al. investigated the combined anti-amyloid and anti-inflammatory effects of liposomes that co-encapsulated microRNA-101 and curcumin in a cell-based model of AD. This formulation effectively targets amyloid precursor protein expression and inflammatory cytokines, suggesting a powerful combination therapy [126]. In a separate study, mesoporous silica NPs containing curcumin, combined with chitosan and Poloxamer-407 hydrogel, enhanced drug permeation and cognitive abilities in a mouse model of AD [127]. Chen and his team developed hydrogels that respond to pH and temperature, integrating the neuroprotective compound timosaponin BII for addressing AD. This hydrogel features ion-sensitive deacetylated gellan gum, thermo-sensitive Poloxamer-407, and sodium alginate, facilitating a quick sol–gel change initiated by heat and calcium ions in the nasal cavity. Research on mice showed enhancements in memory and cognitive function, along with decreased neuroinflammation [128].

Enzyme-labile linkers, such as peptide, ester, and disulfide bonds, incorporated into polymer matrices, can undergo cleavage only in the presence of specific enzymes. This feature allows for on-site activation of the therapeutic components [103]. Although direct examples in AD are still emerging, extensive preclinical studies in cancer nanomedicine provide a strong foundation for applying these concepts. Dutta Gupta et al. described MMP-responsive mesoporous silica–polymer hybrid systems, which release drugs or imaging agents in environments rich in matrix-degrading enzymes. Since similar MMP activity has been observed in AD-related neuroinflammation, these systems are readily adaptable for targeted amyloid-beta clearance or anti-inflammatory therapy [129].

Nanozymes, NPs with enzyme-mimetic properties, serve dual functions: they respond to stimuli for therapeutic action and scavenge ROS. For example, cerium oxide and Fe₃O₄-loaded polymeric carriers mimic the activity of superoxide dismutase, catalase, and peroxidases, neutralizing free radicals while releasing encapsulated antioxidants or inhibitors (RR-11a) [130]. These systems directly target oxidative stress, a key driver of synaptic dysfunction and amyloid-beta aggregation in AD. The study by Mi et al. explores a novel approach for treating AD by using ultrasound-responsive nanobubbles (NBs) loaded with the asparagine endopeptidase (AEP) inhibitors. The NBs were modified with AEP-targeted peptides, allowing them to effectively penetrate the BBB upon ultrasound stimulation. The modified NBs demonstrated strong drug loading and echogenicity. When administered to an APP/PS1 mouse model, the combination of NBs improved the accumulation of inhibitors in the brain, reduced tau cleavage and amyloid plaque deposition, and significantly enhanced cognitive function [131]. Nance et al. explored a non-invasive method that utilizes magnetic resonance-guided focused ultrasound (MRgFUS) MBs and polymeric NPs as therapeutic agents capable of penetrating brain tissues. Coating brain-penetrating NPs with PEG enhances circulation time and stability. Nevertheless, while PEGylation increases NPs’ interactions with cells, it restricts cell uptake and limits their ability to cross the intact BBB. MRgFUS can enhance the concentration and distribution of brain-penetrating NPs in targeted brain regions. The findings indicated that the combination of FUS and MBs could transport 60 nm PEGylated NPs into the brain tissue, exhibiting a 10-fold slower diffusion in normal rat brains. This approach indicates potential for improving efficacy, minimizing side effects, and ensuring sustained drug delivery for various CNS-related disorders, particularly AD [28]. Recently, using FUS/MBs to open the BBB has been proven to enhance the delivery of mRNA-encapsulated lipid NPs (mRNA-LNPs) into the brain. A study showed that by employing FUS/MBs, the delivery of plasmid DNA and the expression of an exogenous protein (luciferase) via mRNA-LNPs in microglia and CD31-positive endothelial cells were observed, which confirms the successful delivery of NPs through the opened BBB [132].

Exogenous triggers such as NIR light, heat, ultrasound, and electric fields have activated liposomal release or disrupted amyloid-beta plaques in preclinical models. These exogenously activated systems enable non-invasive, localized treatment administration. Low-intensity FUS has been shown to temporarily open the BBB and enhance liposome penetration. In a noteworthy study, FUS-assisted delivery of PEGylated liposomes carrying glial-cell-derived neurotrophic factor (GDNF) restored memory function and reduced amyloid-beta deposition in an AD model [133]. Shi et al. developed NIR-absorbing gold nanocages that trap a chelator for metal ions like Cu^2+^, contributing to amyloid fibrillation and oxidative stress. These gold nanocages, functionalized with phenylboronic acid and human IgG, facilitate the controlled release of clioquinol in response to oxidative stimuli. Measuring 50 nm with a negative surface charge, they can cross the BBB. Increased amyloid-beta aggregation elevates H_2_O_2_ levels, breaking the arylboronic ester interaction. NIR light also induces local heating, releasing clioquinol that chelates Cu^2+^, aiding in the dissolution of amyloid-beta plaques and reducing H_2_O_2_ production [134].

When incorporated into polymer carriers, superparamagnetic iron oxide NPs (SPIONs) can be guided via external magnets to target specific regions, facilitating site-selective accumulation. Abbas et al. utilized SPION-loaded, chitosan-coated bilosomes embedded in mucoadhesive wafers to deliver resveratrol intranasally. Under magnetic guidance, these nanocarriers enhanced central nervous system localization, mitigating neuroinflammatory and amyloidogenic markers in lipopolysaccharide-induced AD mice [135]. Hao and colleagues developed thermos-responsive micelles based on conjugated polymers, demonstrating a strong ability to bind lethal amyloid-beta aggregates at physiological temperatures [136]. This approach highlights the synergy between biological and physical targeting mechanisms in achieving precision nanotherapy for AD.

Carbon dots (CDs) were used to interfere with the aggregation of amyloid beta and to inhibit its formation [137]. When equipped with DNA aptamers, these CDs specifically attached to amyloid-beta peptides and were engineered to absorb red light, which enables deeper tissue penetration compared to traditional UV light. Exposure to a 617 nm LED (10 mW cm^−2^) produced ROS, which diminished the toxicity of amyloid-beta peptides and safeguarded neural cells. In vivo experiments indicated that the stereotaxic administration of Apta-CDs reduced aggregates in brain areas affected by amyloid-beta, facilitated by fluorescence-guided therapy. These CDs penetrated brain tissues effectively and offered targeted action for removing amyloid-beta aggregates. Furthermore, CDs modified with the peptide CLIKKPF display adaptability by interacting with phosphatidylserine in atherosclerotic foam cells [138]. Like CDs, fullerenes (C60) have also been explored for their potential against AD. These C60 NPs are employed for their ability to generate ROS when exposed to irradiation and to scavenge ROS in the absence of light. When combined with upconversion NPs and a peptide-targeting amyloid-beta known as KLVFF, the C60 particles could specifically target the aggregates in the AD model of C. elegans. Under NIR light, they facilitated the photooxygenation of amyloid, inhibited the aggregation of 1-methyl-4-phenyl-1,2,3,6-tetrahydropyridine, and reduced cytotoxic effects. Furthermore, in the absence of light, these particles effectively lessened the oxidative stress conditions associated with AD, contributing to an increase in the lifespan of the C. elegans worms [139].

**Table 1:** Strategies for stimuli-responsive DDS for treatment of AD.

Stimuli	Responsive Part	Main Components/Materials	Test Models	Stage	Ref.
pH	Carboxylic group	Pullulan acetate	Ex vivo	Preclinical	[107]
pH	DPPC, DOTAP, Cholesterol	DPPC, DOTAP, Cholesterol	In vivo	Preclinical	[119]
ROS	Boronic ester	MeO-PEG-NH_2_, alkynyl phenylboronic ester	In vitro/in vivo	Preclinical	[108]
ROS and NIR	Boronic ester, Au nanocages	Au nanocages, 4-carbonylphenylboronic acid,	In vitro	Preclinical	[134]
US	Octafluoropropane	PEG, COOOH—modified polystyrene, PLGA, octafluoropropane, human serum albumin	In vitro	Preclinical	[28]
US	Perfluoropropane	DOPC, Cholesterol, DMG-PEG2000, perfluoropropane	In vitro	Preclinical	[132]
US/Enzyme	Octafluoropropane, RR11-a, AAN, RGD	DPPC, (biotinylated DSPE-PEG (2000), RR11-a, octafluoropropane, Biotin-AAN and Biotin-RGD	In vivo	Preclinical	[131]
Temperature	Poloxamer	Poloxamer 407, Chitosan, CTAB	In vivo	Preclinical	[127]
Magnetite	Fe_3_O_4_	FeCl_3_, FeO_4_S, Chitosan, Cholesterol	In vivo	Preclinical	[135]
pH and Temperature	Poloxamer, Gellan gum	Poloxamer 407, Gellan gum, Sodium alginate	In vivo	Preclinical	[128]
NIR, VIS/UV light	Fullerene (C60), NaGdF4:Yb/Er/Tm	Fullerene (C60), NaGdF4:Yb/Er/Tm nanoparticles, KLVF peptide	In vitro	Preclinical	[139]

1,2-dipalmitoyl-sn-glycero-3-phosphocholine (DPPC); 1,2-dioleoyl-3-trimethylammonium-propane (DOTAP); 1,2-dioleoyl-sn-glycero-3-phosphocholine (DOPC); 1,2-dimyristoyl-rac-glycero-3-methoxypolyethylene glycol-2000 (DMG-PEG2000); 1,2-distearoyl-sn-glyc-ero-3-phosphoethanolamine-N-[biotinylated(polyethylene glycol)-2000] (biotinylated DSPE-PEG 2000); Cetyltrimethylammonium bromide (CTAB).

### 3.2. Stimuli-Triggered Nanotherapeutics for Parkinson’s Disease

Parkinson’s disease (PD) is a progressive neurodegenerative disorder that primarily affects the dopaminergic neurons in the substantia nigra [7]. It is characterized by the selective degeneration of these neurons and the abnormal accumulation of misfolded alpha-synuclein aggregates. The condition is further complicated by factors such as mitochondrial dysfunction, oxidative stress, neuroinflammation, and proteasomal impairment. Together, these issues create a complex microenvironment that poses significant challenges for conventional drug therapies [140]. Levodopa (L-DOPA) is the primary treatment for PD. However, it has limitations, including rapid metabolism in the body, inconsistent availability in the brain, and long-term motor complications [141]. Nonetheless, emerging strategies that utilize nanotechnology and stimuli-responsive elements in nanocarriers present a promising approach for developing more effective, targeted, and controlled therapies for PD (Table 2).

Elevated levels of ROS in PD act as important internal triggers for drug release. ROS, including superoxide, hydroxyl radicals, and hydrogen peroxide, are overproduced due to mitochondrial dysfunction, dopamine metabolism, and neuroinflammatory processes. Several ROS-responsive nanocarriers have been developed to leverage this characteristic of PD pathology for site-specific, stimulus-triggered drug release. For example, Lei et al. created ROS-responsive polydopamine–curcumin NPs modified with the RVG29 peptide, enhancing their ability to target the brain. These NPs release curcumin in response to pathological levels of ROS (e.g., H_2_O_2_), providing antioxidant effects and inhibiting the aggregation of alpha-synuclein. In studies involving PC12 cells and MPTP-induced PD mouse models, these NPs reduced ROS levels, prevented the loss of dopaminergic neurons, and improved motor function, as shown by the rotarod, pole test, and swimming performance. Further experiments in C. elegans models confirmed the system’s effectiveness in scavenging intracellular ROS and reducing alpha-synuclein inclusions [142]. Similarly, Fan et al. developed a vehicle-free nanodrug system using a dopamine-thioketal-dopamine dimer combined with rasagiline mesylate. The thioketal linker cleaves in the presence of ROS, releasing the active agents specifically in affected neurons. This nanodrug demonstrated hierarchical targeting through dopamine-transporter-mediated uptake and redox-triggered release, achieving simultaneous dopamine replacement, neuroprotection, and inhibition of neuroinflammation and alpha-synuclein fibrillation in PD mouse models [143]. Lipid-coated polydopamine NPs are recommended for use as antioxidants, neuroprotective agents, and photothermal agents in treating PD. They serve as a platform for photothermal conversion, raising intracellular temperature and enhancing Ca^2+^ influx in SH-SY5Y models when stimulated by NIR light. The NPs showed a notable increase in temperature that depended on their concentration and was linked to the power density of the 808 nm laser, resulting in temperature rises of up to 35 °C [144]. Furthermore, PEGylated polydopamine-coated gold NPs were created for NIR-II stimulation of neurons in rats, which were functionalized with an anti-TRPV1 antibody for targeted delivery, enabling non-invasive photothermal activation of neurons located as deep as 5 mm beneath the cortex [145].

Another promising strategy involves cerium oxide (CeO_2_) NPs, which exhibit intrinsic ROS-scavenging properties due to their ability to transition between Ce^3+^ and Ce^4+^ states [146]. Pichla et al. investigated pH-responsive redox poly(ethylene glycol)-b-poly [4-(2,2,6,6-tetramethylpiperidine-1-oxyl)aminomethylstyrene] NPs (referred to as NRNPs pH) and their effects on the viability of SH-SY5Y cells when treated with 6-OHDA at pH levels of 6.5 and 7.4. These NPs are designed for selective action in lower pH environments. In an aqueous solution, they form self-assembling polymeric micelles that disintegrate in acidic conditions due to the protonation of amino groups present in their core. The study found that pretreatment with NRNPs pH increased cell survival in a concentration-dependent manner only at pH 6.5, while no such effect was noted at pH 7.4 [147].

Additionally, nanogels and hydrogels that respond to temperature and pH fluctuations provide sustained release of therapeutics and improved mucosal adhesion. These advanced delivery systems offer spatiotemporal control, essential for addressing the varied needs of PD treatment while minimizing systemic side effects. Bardajee et al. developed a multi-stimuli-responsive nanocomposite hydrogel made from poly(N-isopropylacrylamide) (PNIPAAm), κ-carrageenan, and Fe_3_O_4_ NPs. This hydrogel demonstrated thermo-responsive and pH-sensitive characteristics and was responsive to magnetic fields. At a physiological pH of 7.4, the hydrogel released approximately 84% of the L-DOPA over 11 days, indicating its potential for prolonged drug delivery. Incorporating Fe_3_O_4_ NPs allowed for the modulation of drug release through external magnetic stimuli, enabling patient-specific dosing adjustments. Tan et al. created a thermosensitive PNIPAAm hydrogel incorporating magnolol nanocrystals (MAG-NCs) for intranasal delivery. The hydrogel remains in a sol state at room temperature but transforms into a gel at body temperature (32 °C), creating a depot within the nasal cavity. This transformation enhances drug retention and facilitates transport to the brain via the olfactory and trigeminal pathways. The MAG-NC gel formulation significantly reduced dopaminergic neuronal loss and oxidative stress in MPTP-induced PD mice, as demonstrated by behavioral improvements and restoration of mitochondrial function [148]. Ren et al. developed a simple method for creating dopamine-based injectable hydrogels by oxidizing a mixture of quaternized chitosan, gelatin, and dopamine in another innovative approach. These hydrogels exhibited stable mechanical strength and good degradability in vitro. Characterization through FTIR and SEM revealed their structure and morphology. They successfully encapsulated dopamine for PD treatment and metronidazole as an anti-inflammatory, with promising release profiles for sustained drug delivery. Cytocompatibility was confirmed with mouse fibroblast cells, providing a convenient method for long-term drug release in PD therapy [149].

Magnetoliposomes (MLPs) provide dual control over targeting and release mechanisms through magnetic fields and redox sensitivity. Cifuentes et al. synthesized MLPs that encapsulate PEGylated magnetite NPs conjugated with L-DOPA and OmpA—an outer membrane protein that facilitates endosomal escape. These MLPs respond to external magnetic fields, allowing for spatially guided delivery, and they also utilize intracellular GSH levels to trigger the release of L-DOPA in a reduction-sensitive manner. In studies involving SH-SY5Y cells and neurons derived from PD models, these MLPs demonstrated enhanced cellular uptake, reduced ROS levels, preserved mitochondrial membrane potential, and enabled controlled delivery of L-DOPA, showing better performance than free drug formulations [150]. The magnetically and redox-responsive MLPs represent a versatile platform combining physical targeting with biochemical specificity. Their potential for multi-drug loading, BBB penetration, and on-demand release makes them promising candidates for advanced therapeutics in PD.

In an innovative approach, Nam et al. developed dual-stimuli-responsive films that use alpha-synuclein as a scaffold for gold and magnetic NPs. These films exhibited shape deformation when exposed to NIR irradiation due to the photothermal heating generated by the gold NPs. Additionally, they displayed mobility in response to magnetic fields. While this research primarily focuses on applications in soft robotics, it introduces a novel concept in which pathological proteins can serve dual purposes: acting as both therapeutic targets and structural components for stimuli-responsive systems. This dual functionality could potentially enable biosensing and localized therapeutic interventions [151]. Polydopamine-based NIR photothermal-assisted magnesium oxide NPs were used as neuroprotective, gene transfection, and photothermal agents. An in vitro SH-SY5Y model induced by rotenone was used to demonstrate the decrease in alpha-synuclein levels and the alleviation of inflammation. The NPs were administered to MPTP-induced PD mouse models, resulting in the restoration of Nissl granules, reduced brain inflammation, and decreased TH+ neuronal loss [152].

**Table 2:** Strategies for stimuli-responsive DDS for treatment of PD.

Stimuli	Responsive Part	Main Components/Materials	Test Models	Stage	Ref.
pH	Amino group	acetal-PEG-SH, chloromethylstyrene	In vitro	Preclinical	[147]
ROS	Ce^3+^/Ce^4+^ ratio	Cerium nitrate	In vitro/In silico	Preclinical	[146]
ROS	Polydopamine, RVG29	Dopamine hydrochloride, RVG29 peptide, Fmoc-NH-PEG-Mal	In vitro/In vivo	Preclinical	[142]
ROS	Dopamine-thioketal-Dopamine	Dopamine, Thioketal	In vitro	Preclinical	[143]
Temperature	PNIPAAm	PNIPAAm, PVP-K30	In vitro/in vivo	Preclinical	[148]
pH, Temperature, and Magnetism	PAA, PNIPAM, Fe_3_O_4_	PNIPAAm, PAA k-carrageenan, FeCl_3_/FeCl_4_	In vitro	Preclinical	[153]
ROS and Magnetism	Fe_3_O_4_	FeCl_3_/FeCl_4_, soy lecithin, OmpA protein, NH_2_-PEG12-COOH	In vitro	Preclinical	[150]
NIR, Magnetism, and Thermosensitive	Au nanoparticles, Fe_3_O_4_, AA, NIPAAM	Au nanoparticles, FeCl_3_/FeCl_4_, AA, NIPAAM	In vitro	Preclinical	[151]
NIR	MgO, Polydopamine	MgO, Polydopamine, PEG, Lactoferin	In vivo	Preclinical	[152]
NIR	Polydopamin	Polydopamine, mPEG-DSPE	In vitro	Preclinical	[144]

Poly(N-isopropylacrylamide) (PNIPAAm); Polyvinylpyrrolidone K30 (PVP-K30); poly (acrylic acid) (PAA)); poly-N-isopropylacrylamide (NIPAAM).

## 4. Challenges and Future Perspectives

Over the past decade, nanotechnology has rapidly advanced, broadening the types and preparation methods of stimuli-responsive nanomaterials and nanocarriers. These systems are highly promising nanotherapeutics due to their unique advantages. Moreover, researchers have also explored dual or multiple stimuli-responsive systems for more precise disease diagnosis and management of neurodegenerative diseases such as AD and PD. Despite the significant progress in their design, aspects such as drug delivery efficiency, controlled release profiles, and in vivo therapeutic outcomes still remain suboptimal. Our understanding of their therapeutic function and underlying chemical/biological interactions is still rudimentary, hindering their commercialization. The translational potential of these systems, especially the highly complex chemically modified ones, remains a significant challenge.

The complexity of the stimuli-responsive nanocarriers, coupled with challenges in their manufacturing, reproducibility, and quality control, has limited their clinical application. Furthermore, concerns regarding safety for long-term systemic use (due to insufficient degradability or biocompatibility) should also be addressed. We still lack a comprehensive understanding of how these systems interact with the human body, and animal models often do not fully replicate human physiology. To accelerate commercialization, future efforts should prioritize developing simpler, more clinically acceptable stimuli-responsive nanocarriers. Enhancing their specificity for individual patients with conditions like neurodegenerative diseases is crucial to minimize potential risks. Safety and efficacy assessments are essential before these nanocarriers can be used in patients. While preclinical studies have shown remarkable progress, extensive clinical trials are still needed to validate their utility.

Nevertheless, with smart and rational design, stimulus-responsive nanotechnology offers a valuable addition to the tools available for drug delivery and molecular imaging in the field of neurodegenerative diseases, warranting continued research and development.

## 5. Conclusions

Neurodegenerative diseases, such as Alzheimer’s and Parkinson’s disease, cause pathological changes in the affected brain tissues, including changes in pH and temperature, high levels of ROS near amyloid-beta plaques, overexpression of some enzymes, and others. These significant changes provide new opportunities for developing smart stimuli-sensitive drug delivery systems. Neurotherapeutic agents can be incorporated into innovative nanocarriers, designed to respond to internal pathophysiological or external stimuli such as magnetic fields and ultrasound waves. Furthermore, due to the physical–chemical changes, such drug-delivery systems can provide specific and selective controlled drug delivery. However, a better understanding of the stimuli-sensitivity of the newly developed drug delivery systems is needed, and the possible risk of the carriers causing undesired off-target effects on the physiological tissues remains a major challenge. Future efforts will probably be focused on creating innovative systems that can respond to certain specific biomarkers present in the pathological tissues in AD and PD.

## Abbreviations

The following abbreviations are used in this manuscript:

AD	Alzheimer’s disease
AEP	Asparagine endopeptidase
ApoE	Apolipoprotein E
ATX	Astaxanthin
BBB	Blood-brain barrier
Biotinylated DSPE-PEG2000	1,2-distearoyl-sn-glyc-ero-3-phosphoethanolamine-N-[biotinylated(polyethylene glycol)-2000]
CDs	Carbon dots
CNS	Central nervous system
CTAB	Cetyltrimethylammonium bromide
DMG-PEG2000	1,2-dimyristoyl-rac-glycero-3-methoxypolyethylene glycol-2000
DOPC	1,2-dioleoyl-sn-glycero-3-phosphocholine
DOTAP	1,2-dioleoyl-3-trimethylammonium-propane
DPPC	1,2-dipalmitoyl-sn-glycero-3-phosphocholine
FUS	Focused ultrasound
GDNF	Glial cell-derived neurotrophic factor
GSH	Glutathione
LCST	Lower critical solution temperature
L-DOPA	Levodopa
MLPs	Magnetoliposomes
MMP	Metalloproteinases
MRgFUS	Magnetic resonance-guided focused ultrasound
NBs	Nanobubbles
NDs	Neurodegenerative disorders
NIPAAM	poly-N-isopropylacrylamide
NIR	Near-infrared
NPs	Nanoparticles
Nrf2	Nuclear factor (erythroid-derived 2)-like 2
PA	Phosphatidic acid
PAA	Poly (acrylic acid)
PC	Phosphatidycholine
PD	Parkinson’s disease
PEG	Polyetylenglycol
PLA	Polylactic acid
PNIPAAm	Poly(N-isopropylacrylamide)
POEGMA	Poly(oligo(ethylene glycol) methyl ether methacrylate
PVP-K30	Polyvinylpyrrolidone K30
ROS	Reactive oxygen species
SPION	Superparamagnetic iron oxide nanoparticles
TfR	Transferrin receptor
